# Supporting syringe services programs in the initiation and scale-up of vaccine administration: findings from in-depth interviews

**DOI:** 10.1186/s12954-022-00681-x

**Published:** 2022-09-01

**Authors:** Monique Carry, Danae Bixler, Mark K. Weng, Mona Doshani, Emma Roberts, Martha P. Montgomery

**Affiliations:** 1 Division of Global HIV & TB, Centers for Disease Control and Prevention, 1600 Clifton Road, Atlanta, GA 30329 USA; 2 Division of Viral Hepatitis, Centers for Disease Control and Prevention, 1600 Clifton Rd, Atlanta, GA 30329 USA; 3 National Harm Reduction Coalition, 243 5th Ave., PO Box 529, New York, NY 10016 USA

**Keywords:** Vaccination, Needle exchange programs, Drug users, Hepatitis A, Hepatitis B, COVID-19

## Abstract

**Background:**

Vaccine-hesitant persons who inject drugs are at increased risk for several vaccine-preventable diseases. However, vaccination rates among this population remain low. While syringe services programs (SSPs) are places where persons who inject drugs feel comfortable accessing services, few offer vaccination services. This study describes facilitators and barriers to vaccination at SSPs.

**Methods:**

We used convenience sampling to conduct semi-structured, qualitative in-depth interviews with 21 SSPs in the USA from June to August 2021. Interview questions asked SSPs about their perceptions, priorities, barriers, facilitators, and the effects of partnerships and policies on vaccine administration. We used deductive thematic analysis to identify the main themes.

**Results:**

Eight (*n* = 8) SSPs offered vaccinations, and thirteen (*n* = 13) did not offer vaccinations. Most SSPs believed offering vaccination services was important, although addressing SSP participants’ immediate needs often took precedence. Staffing, physical space, and logistical issues were the most common barriers to vaccine administration reported by SSPs, followed by SSP participant-related barriers. Facilitators of vaccine administration included access to a tracking system, partnering with agencies or other organizations providing vaccines, and having a licensed vaccination provider on-site. Partnerships provided SSPs opportunities to expand capacity but could also restrict how SSPs operate. Recommended policy changes to facilitate vaccine administration included subsidizing the cost of vaccinations and addressing restrictions around who could administer vaccinations.

**Conclusions:**

Increasing the availability of vaccination services at SSPs requires addressing the varying capacity needs of SSPs, such as tracking systems, licensed vaccinators, and free or low-cost vaccination supplies. While these needs can be met through partnerships and supportive policies, both must consider and reflect cultural competence around the lived experiences of persons who inject drugs.

## Background

Persons who inject drugs are at increased risk for several vaccine-preventable diseases, including hepatitis A [1], hepatitis B [1, 2], and tetanus [3]; persons with substance use disorder are also at increased risk for severe COVID-19 illness [4–7]. Since 2017, the USA has experienced outbreaks of hepatitis A among persons experiencing homelessness and persons who use injection and non-injection drugs. These prolonged outbreaks have resulted in 44,209 infections, including 27,018 (61%) hospitalizations and 420 deaths as of April 15, 2022 [8]. In the USA, 5017 (46%) of 10,991 persons with hepatitis A and available risk factor information reported injection drug use in 2019 [1]. Cases of hepatitis B in the USA have remained stable at approximately 3000 per year between 2010 and 2019 [1]. In 2019, 631 (35%) of 1780 persons with hepatitis B and with available risk factor information reported injection drug use in the USA [1]. Prevalence of past or present hepatitis B among US-born persons with a history of injection drug use increased from 35.3% (95% CI 24.8, 47.6) during 2001–2006 to 58.4% (95% CI 39.5, 75.1) during 2013–2018 [9].

Despite elevated risks of viral hepatitis among people who use drugs and the availability of effective vaccines, recent studies documented vaccination rates of 32–51% for hepatitis A and 35–56% for hepatitis B among this population [10–12]. Accelerated vaccination schedules and incentives have been used to increase vaccination rates for hepatitis B among persons who inject drugs successfully [13]. During the hepatitis A outbreaks, mobile vans and foot teams have offered vaccinations for persons with risk factors, going into non-traditional sites such as homeless encampments, shelters, or public libraries [14]. Vaccines have also been successfully delivered to persons who inject drugs in trusted settings, such as substance use disorder treatment centers and syringe services programs (SSPs) [15, 16].

SSPs are places where persons who inject drugs feel comfortable accessing services [17–19]; however, despite the great need for access to vaccinations among persons who inject drugs, only 62%, 50%, and 46% of 153 SSPs stated that they offered on-site hepatitis A, influenza, and hepatitis B vaccines, respectively, in a 2020 survey [20]. In a 2021 survey of SSPs (*n* = 105), 44% offered any vaccinations on-site; among those, the most commonly provided vaccines were COVID-19 (91%), hepatitis A (76%), influenza (73%), hepatitis B (70%), and tetanus (57%) [21]. Funding has recently been allocated to strengthen service delivery within SSPs, including delivery of vaccinations [22, 23].

The objectives of this study were to describe the range of existing organizational capacity, partnerships, and policy environments that facilitate the delivery of vaccine services through SSPs and the resources needed for SSPs to initiate or scale up their existing vaccination programs.

## Methods

From June through August 2021, we conducted a multistate, rapid mixed-methods assessment of SSPs to assess barriers and opportunities to offering vaccination services to persons who inject drugs at SSPs. We used two cross-sectional methods: a quantitative survey and qualitative, in-depth interviews [24]. In June 2021, we invited SSPs through email listservs and newsletters to participate in our assessment. Our methods and findings from our quantitative survey are published elsewhere [21]. When enrolling in the quantitative survey, SSPs indicated their willingness to participate in in-depth interviews.

We used purposeful convenience sampling to ensure representation from all ten Department of Health and Human Services (HHS) regions and representation from SSPs with and without a vaccination program. SSP staff were eligible to participate if they were 18 or older, could read and speak English, and worked at the SSP for at least 6 months to ensure familiarity with SSP practices.

We conducted and recorded our in-depth interviews with SSPs using a virtual video platform. We used a semi-structured interview guide to ask SSP staff about their organizational perceptions and priorities around vaccine administration, facilitators, and barriers to vaccine administration, and how partnerships and policies affect their ability to administer vaccinations. Participation was voluntary, and the participating SSP staff member provided written and verbal consent before the interview. Contributing SSP respondents received a $50 electronic gift card as a token of appreciation for their time. Because we collected data on program practices, not individuals, the Centers for Disease Control and Prevention (CDC) determined this study to be public health evaluation not requiring institutional review board review.

All audio recordings from our in-depth interviews were transcribed verbatim and thematically coded using MAXQDA 2020 [25]. First, one coder from the research team developed an initial codebook of high-level codes and corresponding definitions, reflective of key concepts from the interview guides [26]. Next, two transcripts, one from an SSP with an on-site vaccination program and one from an SSP without an on-site vaccination program, were coded and used to refine the initial codebook by suggesting additional codes and refining existing codes [27]. Next, four team members met to discuss and agree on each code’s utility and meaning. After coding was complete, all research team members met to discuss consistent response trends and create categories (themes).

## Results

We interviewed 21 SSPs from all ten Health and Human Services regions in the USA (Table 1) [28]. At the time of the interview, eight (*n* = 8) SSPs offered on-site vaccinations (V), and thirteen (*n* = 13) did not provide on-site vaccinations (NV). Our sample's median number of visits to SSPs was 170 (IQR 355–1220) per month, with the median number of unique participants visiting monthly being 109 (IQR 205–572). Eleven (*n* = 11) SSPs were in an urban area, and eight (*n* = 8) were in a rural area. Most SSP locations operated from a fixed site (*n* = 8) or a static site with some mobile delivery (*n* = 8). More than half of our sample (*n* = 12) were operated by a community-based organization, with the remaining operated by a health department (*n* = 8) or substance use treatment or behavioral health center (*n* = 2) (organizations could select multiple response options).

**Table 1:** Characteristic of 21 SSPs participating in interviews, June to August 2021

	Median	Range
*SSP size*		
Visits per month (2 missing)	170	25–1220
Participants per month (6 missing)	109	15–572

	No.	%
*Offers on-site vaccinations*		
Yes	8	38
No	13	62
HHS region* (0 missing)		
1	1	5
2	1	5
3	3	14
4	3	14
5	3	14
6	1	5
7	1	5
8	2	10
9	2	10
10	4	19
*Location (2 missing)*		
Rural	8	42
Urban	11	58
*Model (1 missing)*		
Fixed site only	8	40
Mobile or delivery only	4	20
Both fixed and mobile/delivery	8	40
Operator** (1 missing)		
Community-based organization	12	60
Health department	8	40
Substance use treatment or behavioral health center	2	10

*HHS* Department of Health and Human Services, *SSP* syringe services program

^*^States include California (2), Connecticut (1), District of Columbia (1), Idaho (1), Illinois (1), Kentucky (1), Louisiana (1), Michigan (1), Missouri (1), Montana (1), New York (1), North Carolina (1), North Dakota (1), Ohio (1), South Carolina (1), Virginia (1), Washington (3), West Virginia (1)

^**^Sums to more than total because respondents could select multiple responses

### SSP perceptions and priorities around vaccine administration

To better understand the organizational culture around vaccine administration, we asked SSPs about how staff perceived vaccinations in general. Most SSPs we spoke to supported vaccinations and saw them as consistent with their organizations’ mission and beliefs. One SSP noted the connection between their SSPs' perceptions of vaccines and their ability to encourage vaccinations among their participants. Many participants look to SSP staff and their behavior to determine if vaccines are safe:

"In general, I would say we are pretty pro-vaccine. And we've got a lot of participants who will ask us, 'Have you been vaccinated? What was it like for you? And whenever they ask us, they ask us specifically about COVID vaccines. But they'll ask us about the symptoms we had, like if it hurts getting a shot, or which vaccine we got, and things like that. And they trust us a lot. So, hearing it from us, I have personally been vaccinated. They believe us whenever we tell them things" (V-981).

Any mixed sentiments around vaccination administration were specific to the COVID-19 vaccine. Several SSPs understood that their participants looked to them as a trusted source of information and were hesitant to advocate for a vaccine perceived as new and potentially controversial.

In the context of all the services offered, most SSPs said offering vaccines is an important priority, especially given the populations they serve.

"I think offering vaccinations is part of a whole menu of health care services. I think it ranks up there with other preventative health care services, like PrEP. I would say it ranks high, maybe not at the top. I think our other priorities would be, like I was saying, PrEP, providing wound care, or the buprenorphine clinic. And then probably after that would be the vaccines" (NV-294).

However, while providing vaccines was necessary, some SSPs acknowledged that other priorities often took precedence.

### Challenges and facilitators to vaccine administration

#### Challenges

Despite being a priority, many SSPs recognized specific organizational challenges to vaccine administration. Capacity and logistical issues were the most common barriers to vaccine administration reported by SSPs, followed by participant-related barriers.

“We're trying to do the things we do well, and we don't have the bandwidth to figure this [vaccine administration] stuff out” (NV-387).

Over half the SSPs cited limited capacity as a barrier to vaccine administration. Some capacity issues included not having enough physical space to administer vaccines, inability to store or transport vaccines, and no or limited staff qualified to administer vaccines and complete vaccine-related tasks like data entry, tracking, and follow-up. As one SSP explained, they did not want to advertise a service to participants if no one was available to provide it.

“I think, well, one of the things is, is there a nurse available? Because it's not ideal to talk to somebody and counsel them and get them excited about whatever service it is, including vaccinations. And then, "Oops, nobody's here. You have to come back another time" (V-651).

Most SSPs we spoke to had only one or two full-time staff. Limited staff created the additional problem of time management. Several SSPs struggled with making time to administer vaccines and the associated tasks, such as assessing participants’ needs, accessing records, providing education, data entry, and addressing participants’ vaccination questions. In addition, SSPs with vaccine programs noted that these challenges often created delays or rescheduling of vaccine events, making it tough to offer vaccination services consistently.

“What's been difficult is we've not been able to offer consistency. And I think that's hard. Usually, we like to keep things very consistent, just so our participants know what they can count on” (V-855).

In addition to staffing, many SSPs reported participant-level barriers, including a lack of participant demand for vaccinations and vaccine hesitancy specific to COVID-19 vaccinations, which created challenges for vaccine administration. Several SSPs believed that having more staff would allow them time with participants to stress the importance of vaccinations.

"Honestly, it's just about getting our participants to sit down and discuss that as a need…So I think the biggest barrier is that we don't have that face-to-face interaction with our participants as much as we would like to" (NV-861).

#### Facilitators

We asked the SSPs we interviewed about facilitators for initiating or scaling up vaccination services. Most SSPs not offering vaccine services believe having access to a system to track and look up a participant’s vaccination history would make it easier to offer vaccinations. However, among those SSPs, a couple had concerns about using a vaccine tracking system. For example, one SSP worried about participant acceptance of sharing personally identifiable information linking them to an SSP. Another SSP was unsure if access to such a system would assist their ability to offer vaccines because of their staff's lack of familiarity with navigating a tracking system.

More than half of the SSPs without vaccination programs said they would use a tracking system to verify which patients were vaccinated and who needed vaccinations. Several SSPs said they would use this information to tailor their approaches and create better messaging for participants around vaccinations and other medical needs. One SSP believed such a system would allow them to keep track of some aspects of patients' medical histories, especially those who are unstably housed and often have incomplete or nonexistent medical records.

“Having a tracking system would allow us to create a more targeted approach to who would most likely get a vaccine. And it would probably also help us identify clients who have already been vaccinated” (V-198).

Regarding tracking, we asked SSPs if they believed clients would be concerned about their anonymity. A few thought it could be problematic if the client felt their vaccine record would link them back to an SSP. However, most SSPs did not believe concerns over anonymity to be a substantial barrier, especially if the SSP offered or was co-located with a place that offered other services.

Half of the SSPs without vaccination programs thought partnering with agencies or other organizations currently providing vaccines would help make it easier to start offering vaccinations to their participants. Most SSPs who mentioned partnerships wanted to partner with a medical facility, community clinic, or another SSP. The remaining SSPs desired to partner with local health departments.

About a third of SSPs without vaccination programs believed having a licensed healthcare provider on-site would allow them to initiate a vaccination program. A similar number of SSPs said funding for advertising, health promotion, and infrastructure (e.g., space, storage, refrigeration) would facilitate their ability to start a vaccination program. Other facilitators included staff training and staff buy-in.

"I don't think [our staff is] super comfortable talking about vaccinations or immunizations with our clients just because they don't know much about them… It scares people to talk about it if they're not nurses or don't give the vaccine. So, if they had more information on how to provide or offer them, they could maybe do that" (V-830).

For SSPs currently offering vaccine services, most thoughts increased capacity (e.g., additional staff and expanded hours of operation) would make it easier to scale up their vaccination programs. A few SSPs offering vaccination services mentioned funding to promote vaccination services to the community better and incentivize participants. Example incentives included food, money for transportation, and essential need items (e.g., hygiene items, socks). Staff training to address vaccine hesitancy among participants and community education to combat stigma around SSPs were also described as facilitators.

### Partnerships and policies affecting vaccine administration

Most non-health department SSPs we spoke to have a relationship with their local health department; however, their partnerships ranged from nominal, such as a referral source for participants, to extensive. Almost all SSPs with vaccination programs obtained their vaccines and vaccine-related supplies from the health department. The benefits of partnering with local health departments included access to funding, and physical storage space, staff with clinical training, referrals to needed services, and training opportunities for their staff. Several SSPs explained how their partnerships with the health department allowed them to construct a network of services for their participants.

[The health department] provides all the supplies, the syringes, and safe-to-use supplies. And then, we supply the supplies they cannot purchase with their money. Because we aren't a health department, we can take a few more risks to distribute things like glass pipes or bleach that they can't. So, we come together to provide the items at the SSP and services" (V-911).

One SSP noted that while they were aware of the benefits of partnering with local health departments, some smaller, low-capacity SSPs lack the time and ability to establish these partnerships.

"[F]inding the time to network is very difficult to be able to, 'Hey, this is what we do. We're wondering if we could partner with you in some way'. It's time-consuming. And as far as what we would need is maybe some soft introductions, even if it's just an email introduction to get something going without us having to do our whole spiel, that would be great" (NV-232).

Despite the stated benefits of partnering with local health departments, a few SSPs discussed their concerns with partnering. For example, several SSPs expressed concerns about partnering staff lacking the cultural competence to work with SSP participants and their potential to further stigmatize or mistreat them because of their drug use.

"For the most part, [our relationship with the local health department] is just surface because of what my SSP stands for and why we haven't jumped on some public health grants. First, we are very concerned about harm reduction being co-opted and medicalized. Those with grassroots organizations must ensure that those working in SSPs have lived experience. They know the stigma of different cultural aspects and are familiar with the drug culture and our participants" (NV-374).

Other SSPs were leery that health departments would impose rules around what and how services are offered. One SSP shared how their funding, services provided, and policies were affected by changing administration politics.

"We used to [partner with the local health department], but not so much anymore. The health director changed. And then the sheriff changed, there was someone at the health department who did not like people who use drugs and that caused animosity between her and me, and it ended up dissolving" (NV-561).

During our interviews, we asked SSPs if there are local or state policies or legislation that impacted their ability to provide vaccinations. Almost half of the SSPs identified jurisdiction-specific policies requiring that only a licensed healthcare provider could administer vaccines as a barrier to initiating or scaling up their vaccine programs. One SSP explained how the lack of Medicaid reimbursement for hepatitis A vaccines presented challenges for participants with Medicaid coverage.

“I think just the reimbursement, whatever it is, the Medicaid or Medicare, or both, reimbursement for hepatitis A is a challenge because we only have a limited supply of those free Hep-A vaccines” (V-651).

Several SSPs clarified that negative community attitudes toward SSPs, not policies, limited their ability to advertise for their vaccination program and other services they offered.

“I wouldn't say that any policies make it difficult, especially not at the state level. Locally, I would say our biggest barrier is more attitude towards the SSP program, [and] participants in the SSP program that make it difficult for us to more publicly advertise kind of the services that we're offering” (V-198).

A few SSPs proposed policy or legislative changes to support their vaccination programs. More than one SSP suggested eliminating user fees for all adult vaccinations.

“I think just offering vaccinations for free to all individuals would be the easiest and the most meaningful legislation. It's difficult sometimes to get vaccines to people if they don't fall in certain categories” (V-830).

The remaining proposed policy or legislative changes included removing barriers around who could administer vaccines and getting full reimbursement for hepatitis A vaccines from Medicaid.

## Discussion

SSPs are an essential public health partner for reducing vaccine-preventable diseases among persons who inject drugs. These qualitative interviews with 21 US SSPs identified limited personnel and space capacity, logistical issues, and participant concerns as barriers to vaccination programs at SSPs. Trusted partnerships with organizations providing vaccines, availability of an on-site, licensed vaccinator, and access to a vaccination tracking system were identified as facilitators of vaccination programs at SSPs. Overall, SSPs supported vaccinations and recognized the importance of vaccinations for improving the health of persons who inject drugs; however, SSPs face fundamental challenges in initiating vaccination services for participants. Our interview project identified three opportunities for public health partners to support SSPs in starting or scaling up vaccination services—building capacity for staffing, space, and equipment, providing access to immunization information systems, and enhancing culturally competent partnerships.

To expand vaccination services and provide consistent vaccination access, SSPs need support to increase staffing, space, and equipment capacity. Efforts to increase the number of SSP staff, hire staff who can administer vaccinations, and enable SSPs to provide vaccinations on-site would help make staff more available to engage in vaccine conversations. To address the challenges of hiring licensed vaccinators, jurisdictions could review local regulations and expand the types of providers eligible to administer vaccinations with appropriate training and supervision. Health departments can share existing vaccination training resources with SSPs, which would make it easier for SSP staff to engage participants in vaccine conversations. Addressing participants’ competing priorities by offering food, transportation, or housing resources and reducing the steps needed to receive vaccination would help participants engage in meaningful vaccine conversations. However, understanding the feasibility and effectiveness of direct incentives, like financial incentives, deserves further study. CDC supports SSPs through cooperative agreements to support [29–31] COVID-19 vaccination efforts and, more broadly, expand harm reduction services [32].

Our findings highlighted heterogeneity in the provision of on-site vaccination services. Accessibility of vaccinations depended on the hours of operation, the number of staff, and whether licensed vaccinator staff were available part-time or full-time. SSPs noted the importance of consistency when offering services, including vaccination services. Provided with adequate staffing resources, SSPs will also need support to incorporate vaccination screening and services into regular workflows. Providing SSPs with access to immunization information systems (IISs) will assist SSPs in tracking vaccination histories and tailoring vaccination services. Some SSPs described reviewing vaccination status before participant visits or using a cover sheet to remind providers to ask about vaccination. Improving access to IISs could help SSPs use limited staff time and improve consistency in offering vaccinations.

Most SSPs used partnerships to create a network of services for their participants but also for their sustainability and growth, providing many SSPs with funding, staff, physical space, training, supplies, and capacity building. The trusted relationship between SSP staff and participants is key to increasing vaccine acceptance among persons who inject drugs. Expanding partnerships between SSPs and public health and healthcare agencies should not compromise trusted relationships between SSPs and their participants. Studies of SSPs [33] during the COVID-19 pandemic found that SSPs can be an essential partner in providing vaccinations. Our work supports the previous finding that SSPs should be involved in vaccination planning to tailor vaccination programs to the specific needs of persons who inject drugs. Federal partners can work with SSPs, health departments, and other partners to improve collaboration and cultural competency and reduce the stigma around injection drug use.

This report is subject to limitations. The qualitative interviews were conducted during the first 12 months of COVID-19 vaccine availability; recent COVID-19 experiences informed many responses. Although we interviewed a mixture of SSPs with and without vaccination services and from a wide geographic range, the perspectives in the interviews might not be representative of all SSPs in the USA.

Federal, state, and local public health partners can support SSPs to initiate or scale up vaccination services for persons who inject drugs by supporting the expansion of SSP capacity, providing access to immunization information systems, supporting operational environments conducive to quality partnerships, and addressing the stigma around injection drug use.

## Conclusions

Despite the great need for access to vaccinations among persons who inject drugs, only a few SSPs offer vaccinations on-site. We found that increasing the availability of vaccination services at SSPs requires addressing the varying capacity needs of SSPs, such as tracking systems, licensed vaccinators, and free or low-cost vaccination supplies. Many of these needs can be met through SSPs developing partnerships with existing vaccine providers and supportive policies that lower barriers for SSPs wanting to provide vaccination services. Critical for both success and sustainability, partnerships and policies must consider and reflect cultural competence around the lived experiences of persons who inject drugs.

## Data Availability

The interview transcripts are not publicly available for confidentiality reasons.

## Abbreviations

CDC: Centers for Disease Control and Prevention
COVID-19: Coronavirus disease 2019
HHS: Department of Health and Human Services
IIS: Immunization information systems
NV: No vaccinations (offered)
PrEP: Pre-exposure prophylaxis
SSP: Syringe services programs
V: Vaccinations (offered)
